# Imine- and Amine-Type Macrocycles Derived from Chiral Diamines and Aromatic Dialdehydes

**DOI:** 10.3390/molecules27134097

**Published:** 2022-06-25

**Authors:** Jerzy Lisowski

**Affiliations:** Department of Chemistry, University of Wrocław, 14 F. Joliot-Curie, 50-383 Wrocław, Poland; jerzy.lisowski@chem.uni.wroc.pl; Tel.: +48-71-3757252

**Keywords:** macrocycles, chirality, imines, amines, metal binding, enantiomeric recognition

## Abstract

The condensation of aromatic dialdehydes with chiral diamines, such as 1,2-*trans*-diaminocyclohexane, leads to various enantiopure or *meso*-type macrocyclic Schiff bases, including [2 + 2], [3 + 3], [4 + 4], [6 + 6] and [8 + 8] condensation products. Unlike most cases of macrocycle synthesis, the [3 + 3] macrocycles of this type are sometimes obtained in high yields by direct condensation without a metal template. Macrocycles of other sizes from this family can often be selectively obtained in high yields by a suitable choice of metal template, solvent, or chirality of the building blocks. In particular, the application of a cadmium(II) template results in the expansion of the [2 + 2] macrocycles into giant [6 + 6] and [8 + 8] macrocycles. These imine macrocycles can be reduced to the corresponding macrocyclic amines which can act as hosts for the binding of multiple cations or multiple anions.

## 1. Introduction

The importance of macrocycles in chemistry and biology is well recognized. Among macrocyclic compounds, azamacrocycles constitute an important and diverse class of compounds, which have applications in catalysis, recognition, separation, and medical diagnostics. Perhaps the best-known compounds of the azamacrocyclic class are tetraaza macrocycles, such as porphyrins or 1,4,7,10-tetraazacyclododecane (cyclen) whose derivative 1,4,7,10-tetraazacyclododecane-1,4,7,10-tetraacetic acid (DOTA) is widely used as a Gd(III) complex in magnetic resonance imaging. While tetraazamacrocycles are capable of binding a single metal ion, there are also much larger azamacrocycles containing, for example, 18 nitrogen atoms, which are capable of binding multiple metal ions.

The field of extended macrocycles and their complexes has already been the subject of several review articles, e.g., references [1,2,3,4,5,6,7,8,9,10,11,12,13,14]. In this minireview, extended macrocyclic imines, which are the products of the condensation reactions of aromatic dialdehydes with chiral diamines, such as 1,2-*trans*-diaminocyclohexane (DACH) or 1,2-diphenylethylenediamine (DPEN), as well as the corresponding amine macrocycles, will be described. Particular emphasis will be placed on the ability of these macrocycles to bind guest molecules and their application in enantioselective catalysis. These extended macrocycles in their neutral or deprotonated form are able to bind multiple transition metal ions or large lanthanoid ions. The protonated amine macrocycles of this class are able to bind anions and the neutral forms of these enantiopure macrocycles are also used in enantioselective binding and recognition of chiral organic guest molecules. The properties resulting from the presence of chiral diamine centers, such as chiral recognition and self-recognition or chirality transfer, will also be briefly outlined.

**Figure 1:**
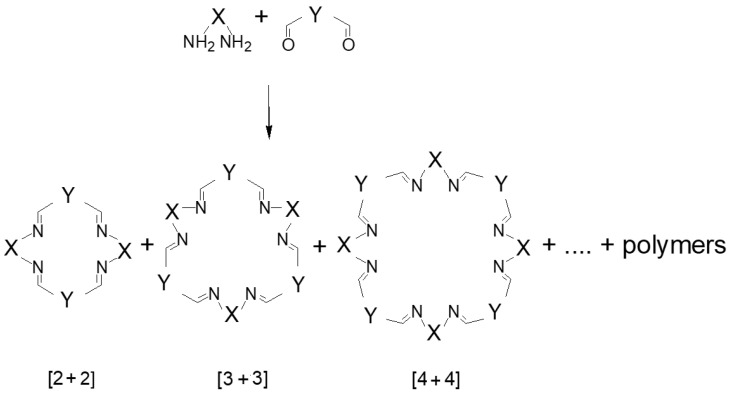
Some of the potential products of the [n + n] condensation of diamines and dialdehydes.

In general, the [n + n] condensation of diamines and dialdehydes may result in the formation of various macrocyclic products, such as [1 + 1], [2 + 2], [3 + 3], etc., macrocycles (Figure 1), as well as oligomeric and polymeric imines. In most cases (especially in the case of saturated substrates) such condensation reactions result in intractable mixtures of mostly polymeric products, and the isolation of pure macrocycles even under dilute conditions is not possible. Nevertheless, in the case of reactions of relatively rigid aromatic dialdehydes with chiral diamines, this type of condensation often leads to the successful preparation of [n + n] macrocyclic products in good yields. Sometimes macrocycles are practically the sole product of such a condensation reaction, and the yields are quantitative. Most typical n values in these reactions are 2, 3 or 4, but in the case of extended dialdehyde building blocks, where two aromatic moieties bearing aldehyde functionalities are connected by a sufficiently long link, [1 + 1] products are also possible.

It should be noted that the formation of the imine bond is reversible [15,16,17,18,19,20,21,22] and that the formation of the various products presented in Figure 1 corresponds to dynamic covalent chemistry. Occasionally, the isolated pure imine macrocycle equilibrates back in solution into a mixture of products. The dynamic library of imines can be transformed into the corresponding mixture of amines by the reaction with reducing agents, such as sodium borohydride, and the corresponding “frozen” library of macrocycles can be separated into individual components. The preferred formation of any given imine [n + n] product may result from the geometric constraints encoded in the building blocks which lead to a preferred geometry of the final thermodynamic product in solution. The equilibrium of the system may also be shifted towards a particular product by its lowest solubility and crystallization. Finally, the equilibrium among the [n + n] imines may be shifted by metal ion templates towards the macrocycle which is best suited for metal complexation [23,24].

## 2. [2 + 2] Macrocycles

**Figure 2:**
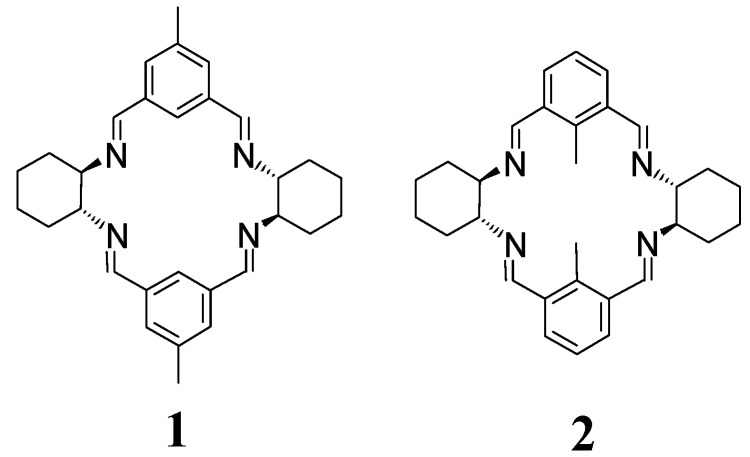
[2 + 2] macrocycles obtained from the kinetic [3 + 3] products.

Condensation of isophthalaldehyde and its derivatives with enantiopure DACH leads typically to a mixture of [3 + 3] and [2 + 2] macrocyclic products where the larger [3 + 3] macrocycles are the dominant kinetic products, while the [2 + 2] macrocycles seem to be the thermodynamic products. Thus, prolonged reflux of [3 + 3] macrocycles in dichloromethane (DCM) resulted in the formation of smaller [2 + 2] macrocycles, such as **1** and **2** in quantitative yields (Figure 2) [25].

**Figure 3:**
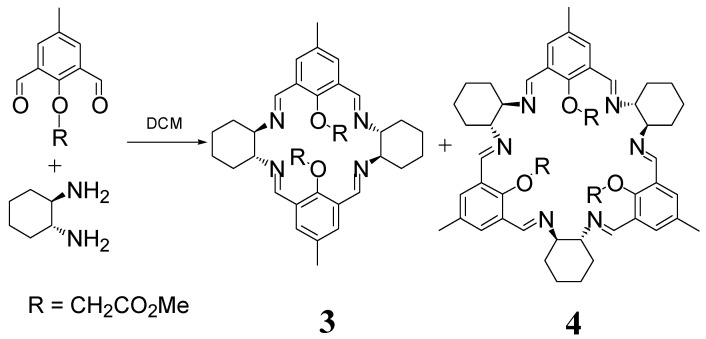
Formation of a mixture of [2 + 2] and [3 + 3] products.

**Figure 4:**
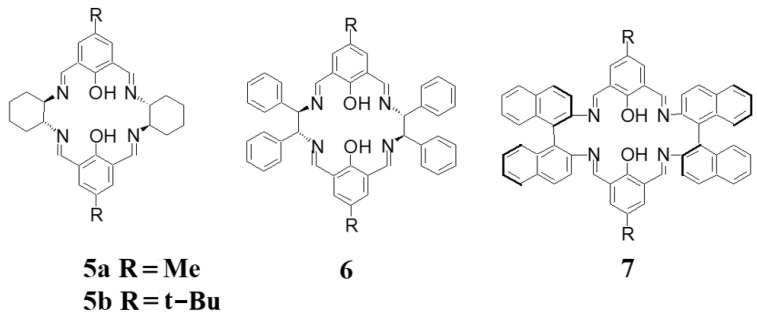
[2 + 2] diphenolic imines obtained in metal-templated condensations.

The [2 + 2] macrocycle **3** is also accompanying the main [3 + 3] macrocycle **4** (which can be isolated at only a 6% yield as a pure product) in the case of the condensation of DACH with an O-alkylated 2,6-diformylphenol (Figure 3) [26]. In contrast, the [2 + 2] macrocyclic imines **5**–**7** (Figure 4) derived from chiral diamines and 2,6-diformylphenols can be obtained in high (typically 50–90%) yields as dinuclear metal complexes of the deprotonated form of the macrocycles in condensation reactions templated by transition metal ions, such as zinc(II), copper(II) or nickel(II) [27,28,29,30,31,32,33,34].

It should be noted that without a metal template the corresponding condensation reactions usually lead to [3 + 3] macrocycles (see Section 3). However, this is not the case when substrates used for **7** are reacted without a metal template. In this system mixture of imines is formed without templating ions. When the lead(II) ions are used as the template, the resulting dinuclear complexes can be reduced by sodium borohydride and after demetallation afford the corresponding free amine macrocycles. The dinuclear complexes of **5a**, **b** and **6** are relatively flat and moderately twisted, while the conformation of the Cu(II) complex of **7** is considerably twisted [34]. Dinuclear zinc(II) complex of **5a**, as well as dinuclear zinc(II) complex of analogous *meso*-type [2 + 2] macrocycle derived from racemic DACH, are fluorescent agents [30]. Moreover, these compounds induced apoptosis of cancer cells and the *meso* complex was found to be an efficient regulator of the cell cycle and anti-apoptosis genes. Dinuclear cobalt(II/III) complexes of **5a** and **6** catalyzed asymmetric cyclopropanation of styrene with diazoacetate with high enantioselectivity reaching up to 94% [31] and the dinuclear copper(II) complexes of **5a**,**b** and **6** and their amine counterparts were applied in enantioselective oxidative coupling of 2-naphthol [32]. The dinuclear zinc(II) complex of **6** was also studied as an enantioselective catalyst for the desymmetrization of meso diol to achieve a chiral product with 96% yield and 88% ee [33]. The dinuclear copper(II) complex of **7** was studied as an enantioselective catalyst in the asymmetric oxidative coupling of 2-naphthol to chiral 1,1′-bi-2-naphthol, which is an important chiral ligand (BINOL) [34].

**Figure 5:**
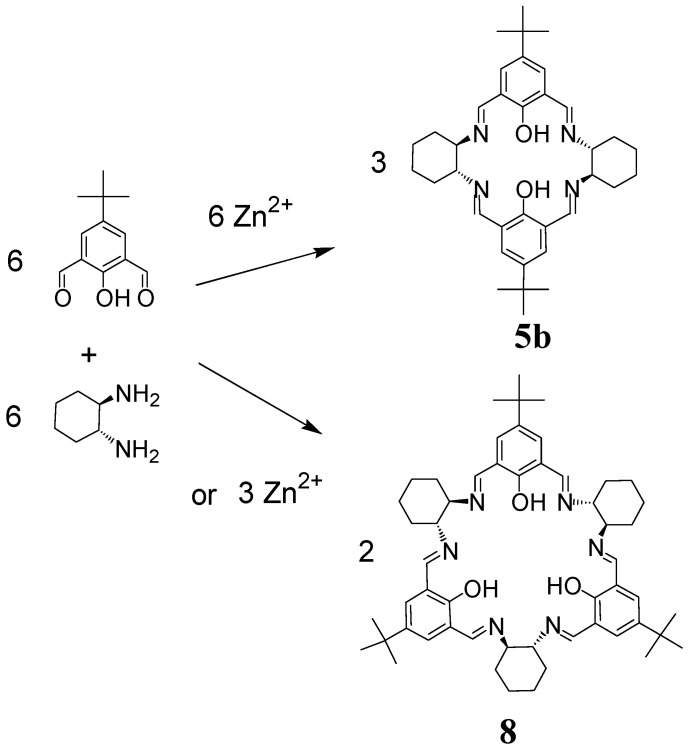
Dependence of the size of the macrocycle on the amount of added template.

In the case of the reaction of 2,6-diformyl-4-*tert*-butylphenol and enantiopure DACH, an unprecedented template effect was observed [27,28]. In this system, the size of the formed macrocycle depends on the stoichiometry of the applied metal template. By using equimolar amounts of dialdehyde, diamine and zinc(II) acetate (Figure 5), the dinuclear Zn(II) complex of the [2 + 2] macrocycle **5b** is selectively obtained. On the other hand, by using just half of the equivalent of the same template metal salt the [3 + 3] macrocycle **8** is selectively obtained in the form of a trinuclear Zn(II) complex where the three metal ions are shared by two deprotonated macrocyclic units.

**Figure 6:**
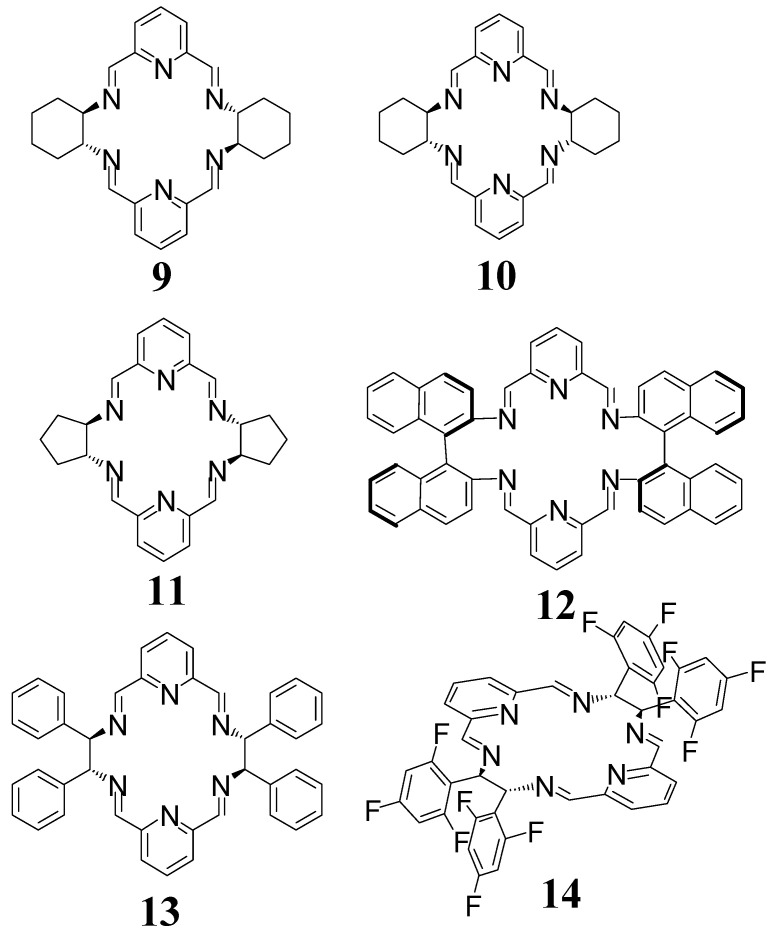
Imine [2 + 2] macrocycles derived from 2,6-diformylpyridine.

The condensations of 2,6-diformylpyridine (DFP) with enantiopure chiral diamines result in mixtures of macrocyclic products and the isolation of pure imine products is unsuccessful, partly due the reversible nature of the condensation reactions. For instance, the reaction of DFP with enantiopure DACH results in a mixture containing mostly the [3 + 3] and [2 + 2] imine macrocycles, but the pure product **9** (Figure 6) was not isolated from this mixture. This dynamic library can be “frozen” by reduction with sodium borohydride and the resulting amine [2 + 2] and [3 + 3] macrocycles can be separated by recrystallization [35]. On the other hand, the reaction DFP with the racemic form of DACH results in a mixture containing mainly isomeric meso-type [2 + 2] macrocycle **10** (Figure 6) and meso-type [4 + 4] macrocycle [36]. Pure imine **10** can be separated from this mixture as the least soluble product.

The chiral [2 + 2] imine **9** can be easily obtained in high yields (typically 40–80% for crystallized products) in the complexed form, when large metal ions, such as lanthanoid(III) or lead(II) ions are used as a template in the reaction of enantiopure DACH [37,38,39,40]. The application of these ions shifts the dynamic equilibrium selectively towards the [2 + 2] product. In most cases, the NMR spectra of the crude reaction mixtures containing these complexes of **9** indicate practically the quantitative formation of this macrocycle. A similar effect was observed in the case of formation of complexes of macrocycle **11** derived from the cyclopentane analog of DACH, that is *trans*-1,2-diaminocyclopentane (DACP) [41]. Another product derived from DFP is the macrocycle **12** based on the enantiopure 1,1′-binaphthyl-2,2′-diamine [42] and macrocycles **13** and **14** derived from DPEN or its fluorinated derivative [43,44,45] (Figure 6). The dysprosium complexes of macrocycles **13** and **14** exhibit exceptional Single Molecule Magnet (SMM) properties with record values of the energy barrier for the reorientation of the magnetization (U_eff_) among air-stable SMMs known so far [44,45]. Interestingly, the application of racemic DACH in a similar templated condensation does not result in the complexes of *meso*-type macrocycle **10** (these complexes may be formed only as intermediate kinetic products) but leads to a racemic form of the complexes of chiral macrocycle **9** as the thermodynamic products.

**Figure 7:**
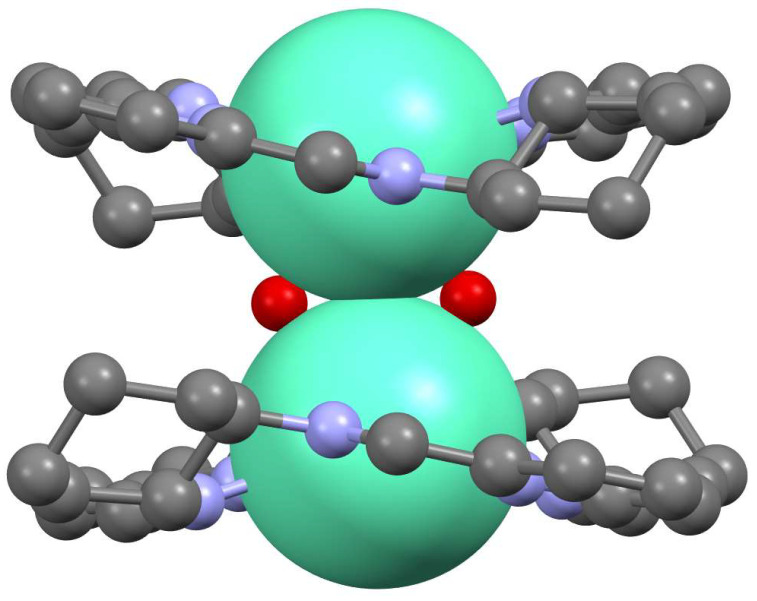
Side view of a dimeric complex where two Eu(III) ions residing in two macrocyclic units of 9 are linked by additional hydroxo bridges (metal ions are shown in spacefill representation, hydrogen atoms and additional anions are omitted for simplicity, Eu—green, N—blue, O—red). All the figures presenting molecular structures in this review were redrawn using the Mercury 2020.1 program and were based on the appropriate cif files deposited at the Cambridge Crystallographic Data Centre.

Macrocycles **9**, **11**–**14** adopt helical conformations in their enantiopure lanthanoid(III) complexes. This conformation leads to interesting properties in these complexes related to their chiral nature, i.e., enantiomeric self-recognition, chirality transfer and enantioselective hydrolytic cleavage of DNA [38,39,40,46]. Two macrocyclic units of **9** containing lanthanoid(III) may be linked by hydroxo or fluorido bridges to form dinuclear complexes (Figure 7) [38,39,40]. These complexes are formed only when both macrocyclic units are derived from the monomeric complexes of the same chirality, i.e., the same direction of the helical twist. This corresponds to enantiomeric self-recognition, which is a narcissistic sorting of macrocyclic units with respect to their chirality. In similar heterodinuclear complexes, where one macrocyclic unit of **9** is linked to the macrocyclic unit of the achiral macrocycle derived from ethylenediamine, the achiral macrocycle adopts a defined direction of helical twist dictated by the direction of the helical twist of **9**. This effect corresponds to chirality transfer between macrocyclic units.

**Figure 8:**
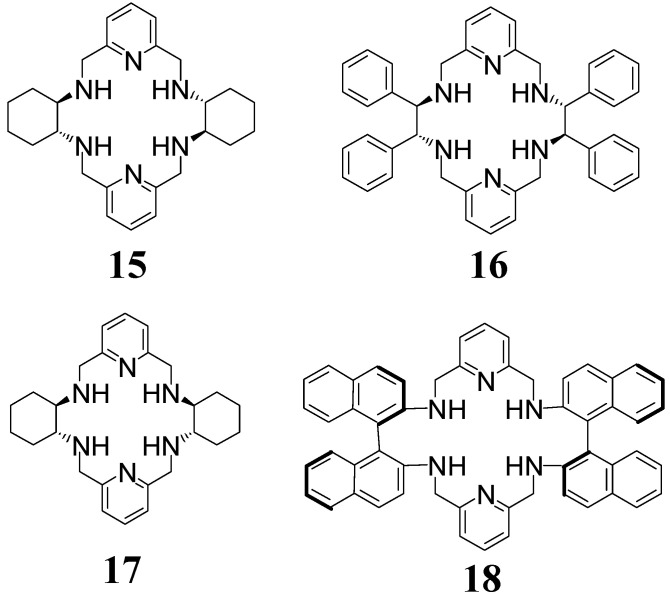
[2 + 2] amines derived from 2,6-diformylpyridine.

Lead(II) complexes of macrocycles **9**, **10**, **12**, and **13** can be reduced and demetallated to give the corresponding free macrocyclic amines **15**–**18** (Figure 8). Unexpectedly, amine **18** does not bind lanthanoid(III) ions, unlike its imine counterpart. On the other hand, macrocycle **15** forms lanthanoid(III) complexes, which can undergo solvent [47] or anion-induced [48,49] helicity inversion. Macrocycle **15** and its derivatives, as well as macrocycles **16** and **18**, can also be used as chiral solvating agents for the enantiodiscrimination of chiral carboxylic acids, such as ibuprofen. The determination of the enantiomeric excess of different carboxylic acids has been achieved on the basis of good splitting of the NMR signals for the enantiomers of the bound guest molecules [50,51,52,53]. Interestingly, in the gas phase, amine **15** is able to bind potassium cations, anions, such as carboxylates, but also to function as an acceptor of contact K^+^/anion pairs [54,55].

**Figure 9:**
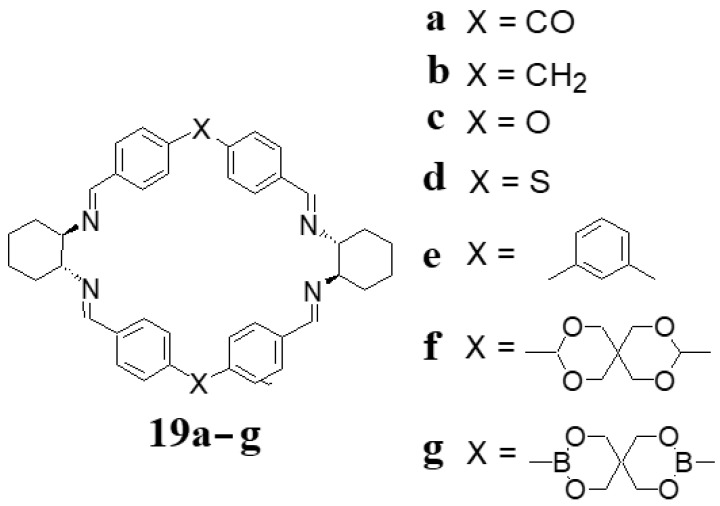
Examples of rhombimine macrocycles.

The formation of [2 + 2] imines is preferred when enantiopure DACH is condensed with dialdehydes based on two benzaldehyde fragments that are connected by a link X (Figure 9) which enforces the bent conformation of the dialdehyde. Because of the resulting shape of the macrocycle, these products were called rhombimines (Figure 9) [56,57,58,59,60]. The reduced form of this kind of macrocycles, i.e., rhombamines was applied in NMR enantiodiscrimination of chiral carboxylic acids and their derivatives [61,62].

**Figure 10:**
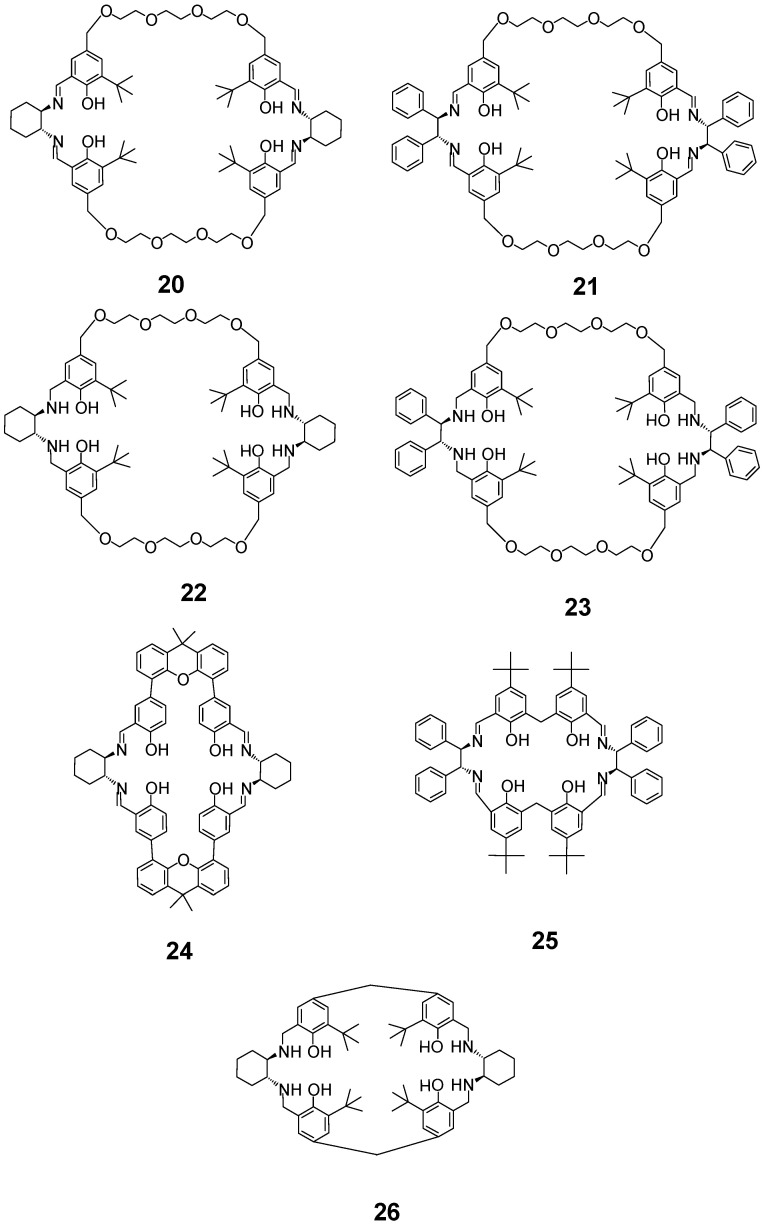
Extended tetraphenolic [2 + 2] macrocycles.

The condensation of enantiopure DACH or DPEN with aromatic dialdehydes containing two phenol fragments results in the formation of the extended [2 + 2] imines **20**, **21**, **24**–**26**, which can also be transformed into their reduced amine forms **22** and **23** (Figure 10) [63,64,65,66,67,68,69,70]. These enantiopure ligands contain two compartments corresponding to the chiral salen environment (the parent macrocycle **26** was called calixsalen [63]). Transition metal complexes of acyclic salen-type ligands are well known to exhibit catalytic activity. Macrocycles **20**–**26** correspond to the “doubled” form of salen/salan ligands, and they adopt a folded conformation in their complexes. These macrocycles allow for the synergistic catalytic activity of two ligand-bound metal centers, as well as provide a kind of pocket for substrate binding. For example, in the dinuclear cobalt(III) complex of **20** one metal center may coordinate a hydroxide nucleophile, while the other metal center can activate epoxide. The energy-minimized structure of such a dinuclear complex shows a plausible intermediate in the catalytic cycle, where the epichlorohydrin molecule is bound in the center of the expanded macrocycle between the two cobalt(III) ions [64]. The extended tetraphenolic macrocycles **20**–**26** are predisposed to form dinuclear complexes with transition metals, such as cobalt(III), copper(II), vanadium(V), manganese(III) or titanium(IV), which exhibit interesting enantioselective catalytic activity in the kinetic resolution of epoxides [64], Henry reaction [65], aza-Henry reaction [70], Strecker reaction [67], O-acetylcyanation/cyanoformylation of aldehydes [66], asymmetric carbonylation of aldehydes [68], and epoxidation of alkenes [69]. The dinuclear copper(II) complex of another tetraphenolic [2 + 2] imine derived from DPEN was active as an enantioselective catalyst in the Henry reaction [71].

**Figure 11:**
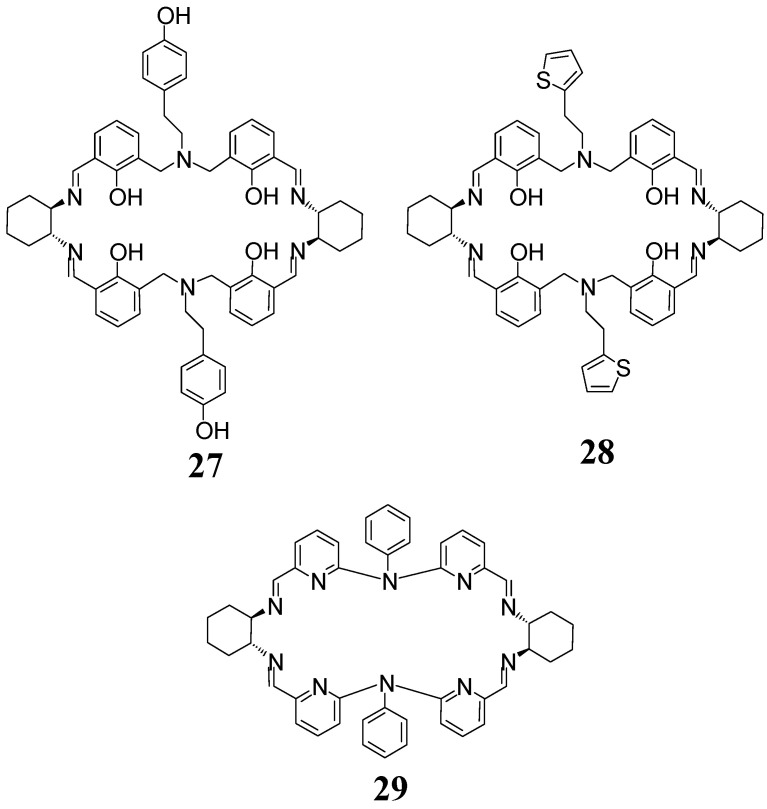
Extended [2 + 2] imines obtained in metal-templated condensations.

In contrast to the above tetraphenolic macrocycles obtained as free ligands, the extended tetraphenolic macrocycles **27** and **28** (Figure 11) were obtained only as metal complexes in condensation reactions templated by zinc(II) ions [72,73]. Similarly, the decaaza macrocycle **29** was obtained in template condensation [74]. In the latter case, an interesting influence of the kind of the metal template on the reaction of enantiopure DACH with the appropriate aromatic dialdehyde was observed. While the application of cadmium(II) salt resulted in the formation of the dinuclear Cd(II) complex of macrocycle **29**, the application of copper(II) salt resulted in the formation of a mononuclear complex of the acyclic condensation product. The dinuclear Cu(II) complex was obtained, however, by transmetalation of the Cd(II) complex and the free macrocycle was obtained by the demetallation of the complexes with sodium sulfide via the formation of insoluble CuS.

**Figure 12:**
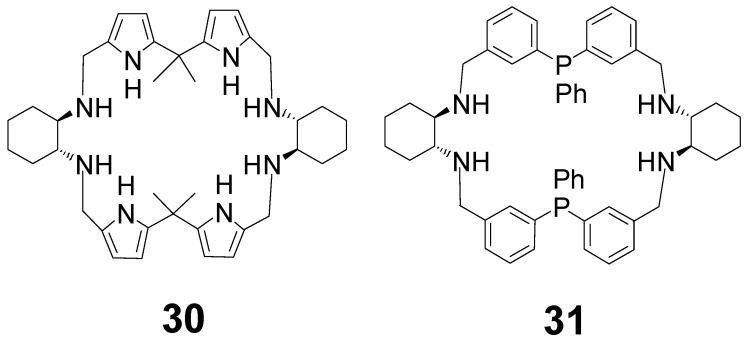
Extended [2 + 2] amine ligands for enantioselective catalysis.

The imine precursor of the extended [2 + 2] amine **30** with four pyrrole fragments (Figure 12) was obtained by the condensation of DACH L-tartarate with dipyrrolic dicarbaldehyde in the presence of triethylamine with a 90% yield. This imine was reduced with sodium borohydride to give the amine macrocycle **30** [75]. The copper(II) complex of **30** was applied as an enantioselective catalyst in a Henry reaction with up to 95% ee values. The iron complex generated from the [2 + 2] amine **31** and triiron dodecacarbonyl was used in the enantioselective hydrogenation of ketones [76,77]. This N_4_P_2_ ligand contains two phosphine-type phosphorous donor atoms which allow the stabilization of the low oxidation state of iron in the active complex.

**Figure 13:**
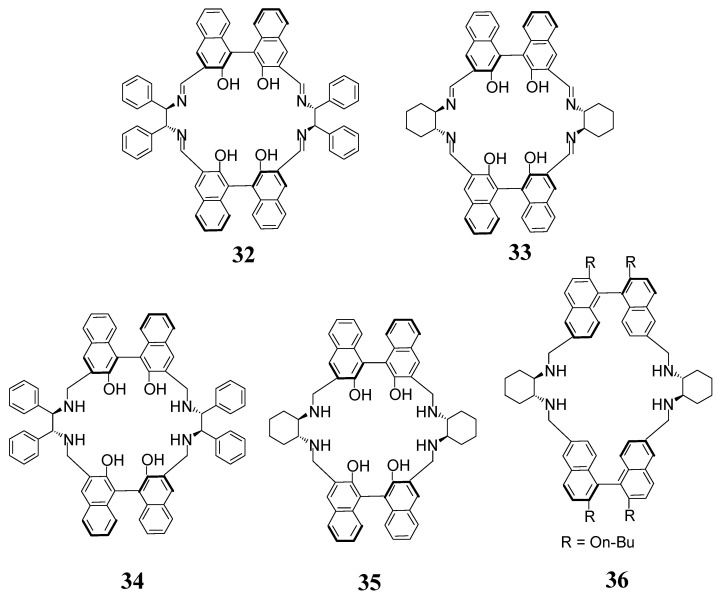
[2 + 2] macrocycles used as fluorescent detectors of guests.

The enantiopure [2 + 2] imine and amine macrocycles **32**–**35** (Figure 13) have two chiral centers—chiral diamine fragment and chiral 1,1′-bi-2-naphthol (BINOL) fragment. These compounds were successfully used in enantioselective fluorescent recognition of mandelic acid and other chiral acids [78,79,80]. Macrocycle **35** was also used as a fluorescent probe selectively recognizing the mercury(II) cations [81]. Similar macrocyclic amine **36** acts as a selective fluorescent sensor for the detection of zinc(II) ions [82] as well as for the combined recognition of copper(II) ions and amino acids [83].

## 3. [3 + 3] Macrocycles

**Figure 14:**
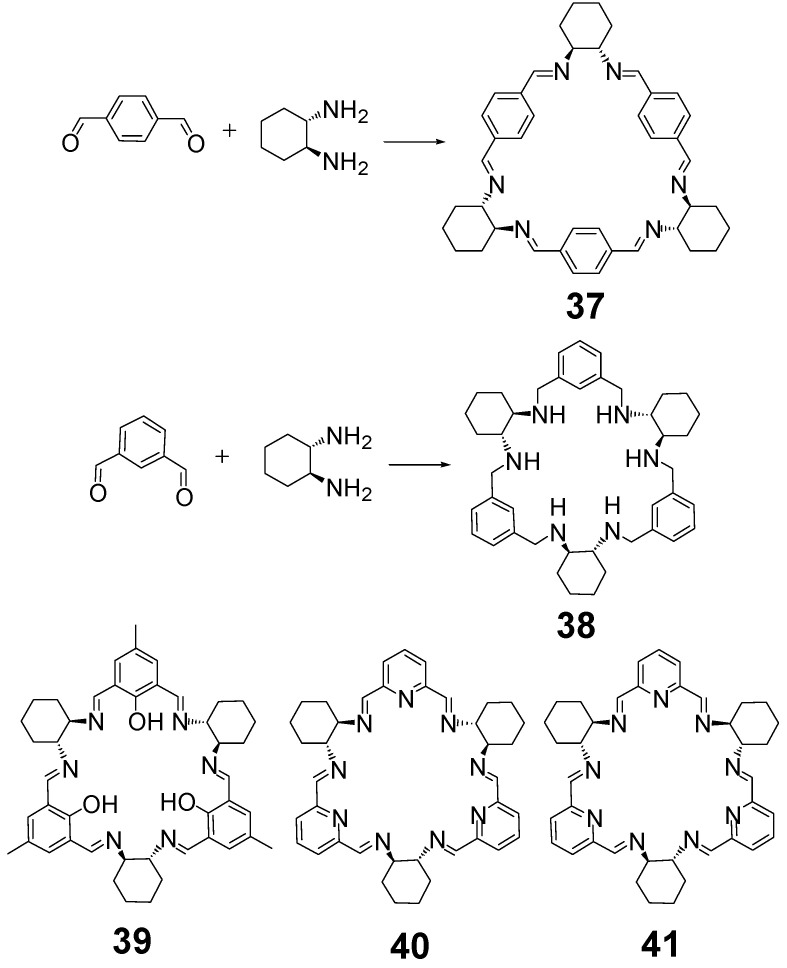
Examples of [3 + 3] imines derived from DACH.

**Figure 15:**
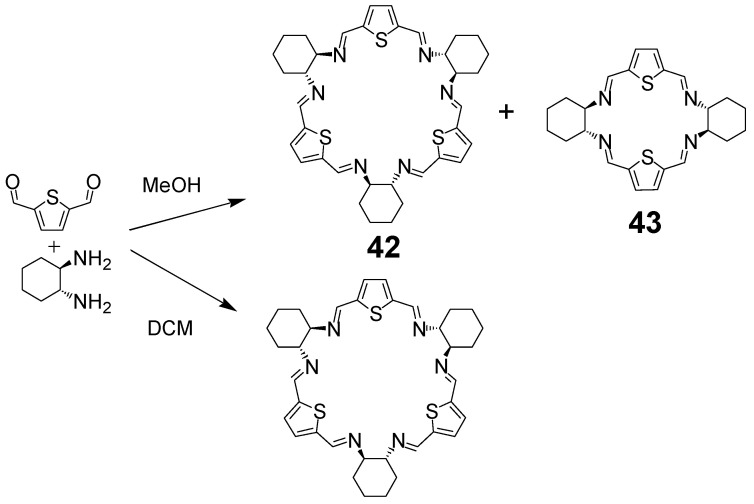
Macrocycles from 2,6-diformylthiophene.

The angle formed by the amine groups of DACH in combination with the linear alignment of aldehyde groups of 1,4-terephthalaldehyde leads to the preferred [3 + 3] condensation product **37** of triangular shape (Figure 14), which was discovered in 2000 by Gawroński et al. and was called trianglimine [84]. This macrocyclic crude product can be obtained in quantitative yield simply by mixing substrates in dichloromethane at room temperature and subsequent evaporation of the solvent, while the yield after recrystallization was ca. 90%. This strong preference for the formation of [3 + 3] products is also observed in reactions of DACH with other linear dialdehydes (with aldehyde groups rigidly positioned at 180 degrees) leading to a rich family of trianglimines. A similar reaction of DACH with 1,3-isophthalaldehyde or its derivatives leads to [3 + 3] products called isotrianglimines, such as macrocycle **4** (Figure 3) or macrocycle **38** (Figure 14). As was already mentioned above, in the case of the condensation of isophthalaldehydes or DFP with enantiopure DACH, the [3 + 3] products **38** and **40** (Figure 14), respectively, are accompanied by [2 + 2] macrocyclic products. A similar situation was observed in the case of thiophene derivatives (Figure 15) where both macrocycles **42** and **43** were formed when the reaction was run in methanol. In contrast, the [3 + 3] macrocycle **42** was the sole product when the reaction was run in dichloromethane [25]. The introduction of the phenol group also influences the condensation reaction, the hydroxyl derivative of isophthalaldehyde, that is 2,6-diformyl-4-methylphenol, reacts with enantiopure DACH in acetonitrile to give macrocycle **39** as the sole product in practically quantitative yield (Figure 14) [85]. These and related macrocycles are called calixsalens and the phenolic macrocycles derived from dihydroxyisophthalaldehydes are called resorcisalens.

The rich family of trianglimines, isotrianglimines, calixsalens and resorcisalens has recently been the subject of an excellent review [8] and will not be discussed in detail. Only a few selected examples with special emphasis on metal derivatives of [3 + 3] imines and amines will be presented here.

**Figure 16:**
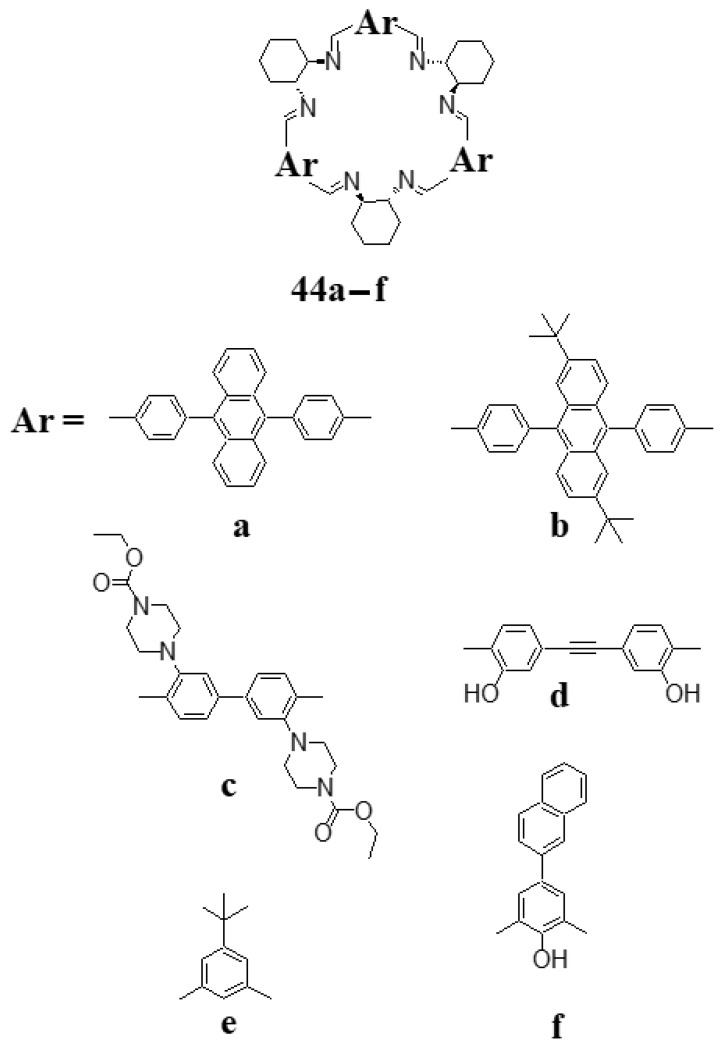
Examples of macrocyclic [3 + 3] imines derived from DACH.

The application of extended rigid linear aromatic dialdehydes leads to expanded triangular [3 + 3] molecules, such as macrocycles **44a–d** (Figure 16). In a recent paper [86] Grajewski et al. described the synthesis of the extended trianglimine **44a** and its amine counterpart, as well as the corresponding endoperoxide derivatives of these macrocycles. Additionally, the reversible cycloaddition of singlet oxygen to anthracene fragments of **44a** without degradation of the macrocyclic system was demonstrated. Olson et al. demonstrated luminescent properties of extended trianglimines, such as **44c** and the corresponding trianglamines [87]. Both theoretical DFT optimized structures and experimental X-ray structures indicate that in this and similar imines the six large substituents appended to the biphenyl legs of the trianglimine macrocycles adopt an alternating conformation. In this conformation, these substituents partly close the space above and below the mean trianglimine plane, which leads to the formation of a kind of container molecule.

The [3 + 3] imines, such as **8**, **39**, **38**, **44e** and **44f** exhibit rich supramolecular chemistry. They can act as hosts for small organic guest molecules, form dimers and capsules or self-associate into larger structures. The formation of various noncovalent aggregates of these macrocycles was demonstrated by Kwit, Janiak and others for solid, liquid, and gas phases [88,89,90,91,92,93,94,95].

**Figure 17:**
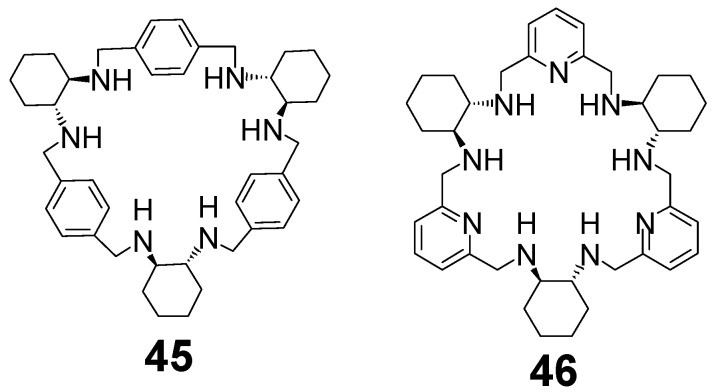
[3 + 3] amine macrocycles.

The reduced form of trianglimine **37** is trianglamine **45** (Figure 17) This macrocycle is able to bind zinc(II) ions and this enantiopure complex generated in situ is an active catalyst in the enantioselective hydrosilylation of imines [96] and ketones. This trianglamine as well as its derivative, where methylene bridges are substituted with additional phenyl rings, functions as an efficient chiral solvating agent in the enantiodiscrimination of chiral carboxylic acids [97,98]. Calixsalene **39** and its derivatives were used in enantioselective recognition of carboxylic acids on the basis of NMR [99,100]. The expanded macrocycle **44d** has three chiral salen-like compartments and is predisposed to form trinuclear complexes [101].

**Figure 18:**
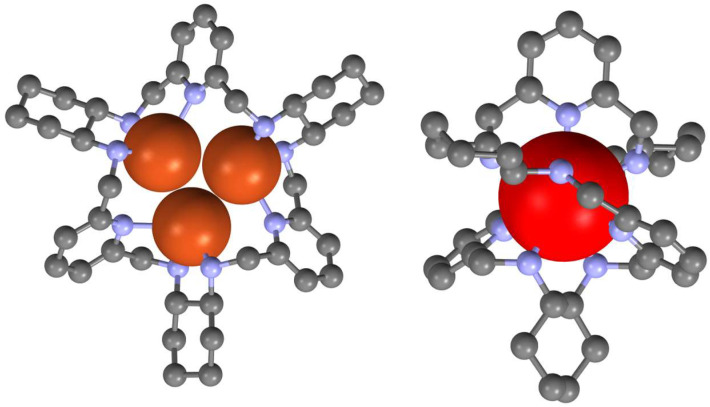
Structures of trinuclear copper(II) (**left**) and mononuclear ytterbium(II) (**right**) complexes of macrocycle **46**. Metal ions are shown in spacefill representation, hydrogen atoms and additional anions omitted for simplicity.

While the metal complexes of [3 + 3] imine **40** derived from DACH and DFP were not isolated in a pure form, the condensation of enantiopure DACH in the presence of cadmium chloride shifts the equilibrium to a dinuclear complex of this imine, which can be reduced and demetallated to the corresponding amine **46** (Figure 17) [102]. Similarly, when racemic DACH is used in this condensation reaction, the cadmium(II) template shifts the equilibrium of imine products into a heterochiral [3 + 3] imine **41**, which can subsequently be reduced to heterochiral amine [103]. The amine **46** is able to bind two or three transition metal ions [104,105] or a single lanthanoid(III) ion [35,106,107]. In the latter type of complex, the large macrocycle wraps around the metal ion to form a double-helical conformation (Figure 18). A rare process of helicity inversion of **46** between the kinetic and thermodynamic complexation product was observed for these complexes. A similar process was observed in the case of complexes of heterochiral RRRRSS analog of **46** derived from imine **41** [108].

**Figure 19:**
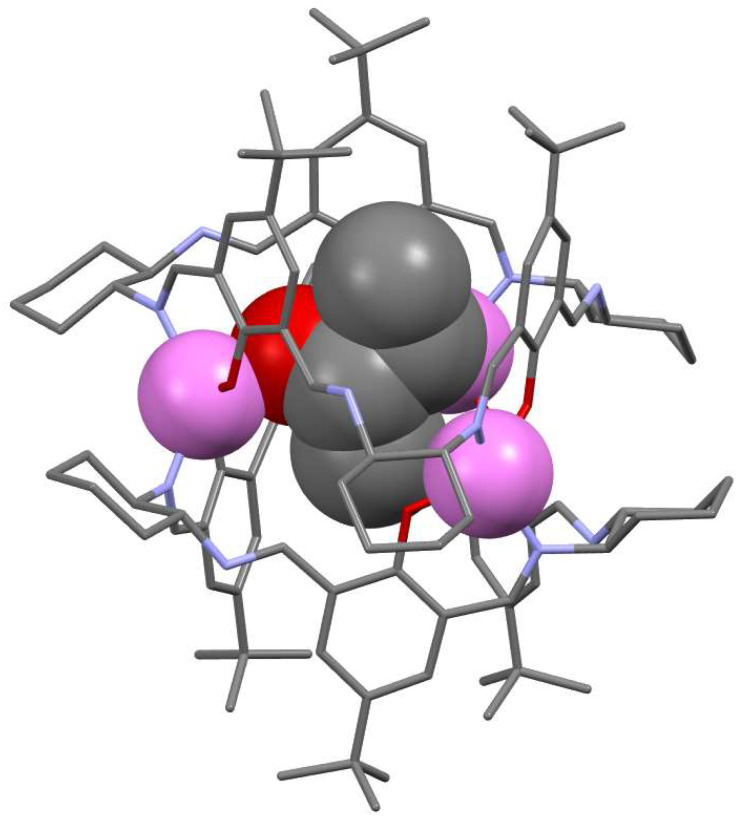
Metal-organic cage based on two macrocyclic units of **8**. Zinc atoms (violet) and (*S*)-2-butanol are in spacefill representation.

The reactions of [3 + 3] triphenolic calixsalen macrocycles, such as **8** and **39** or similar imines with metal salts do not lead to complexes of these macrocycles but to complexes of the contracted [2 + 2] forms or acyclic ligands [23,24,109]. For instance, the reaction of **8** or **39** with an excess of zinc(II) acetate leads to dinuclear complexes of **5b** and **5a**, respectively. In contrast, when **8** is reacted with zinc(II) acetate in a 2:3 molar ratio, a metal-organic cage complex of deprotonated macrocycle [Zn_3_**8**_2_] is formed (Figure 19) [27,28]. This barrel-shaped molecule has an empty interior. This cavity may be occupied by solvent molecules or gas molecules which results in remarkable gas sorption properties for some crystalline forms. Moreover, this chiral complex exhibits enantioselective binding of small guest molecules, such as 2-butanol (Figure 19). The ability to bind guest molecules and the enantiopure nature of [Zn_3_**8**_2_] was the basis of its application in the enantioseparations of chiral organic compounds by using gas chromatography [110] or capillary electrochromatography [111,112].

**Figure 20:**
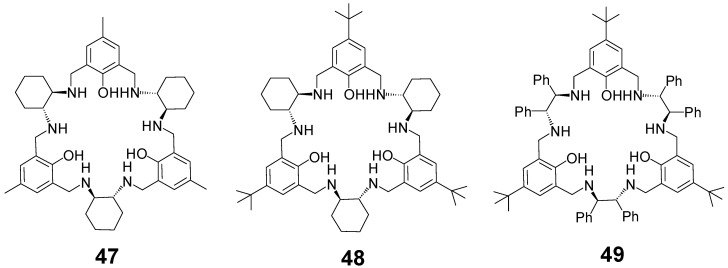
Amine counterparts of calixsalenes.

In contrast to the [3 + 3] calixsalene imine macrocycles, such as **39**, their amine counterparts, such as **47**–**49** (Figure 20) easily form trinuclear complexes with transition metals [113,114]. In zinc(II) complexes of this type, a synergistic enantioselective effect of the three metal centers was observed in catalytic asymmetric aldol and Henry reactions [115]. These macrocycles are also able to bind one, two or three larger lanthanoid(III) ions in their cavities [116,117,118,119,120]. In these complexes, a trinuclear lanthanoid dihydroxo cluster is bound in the center of the macrocycle. The metal ions in these lanthanoid(III) complexes are additionally linked by phenoxo, as well as hydroxo bridges, which are associated with their magnetic properties.

**Figure 21:**
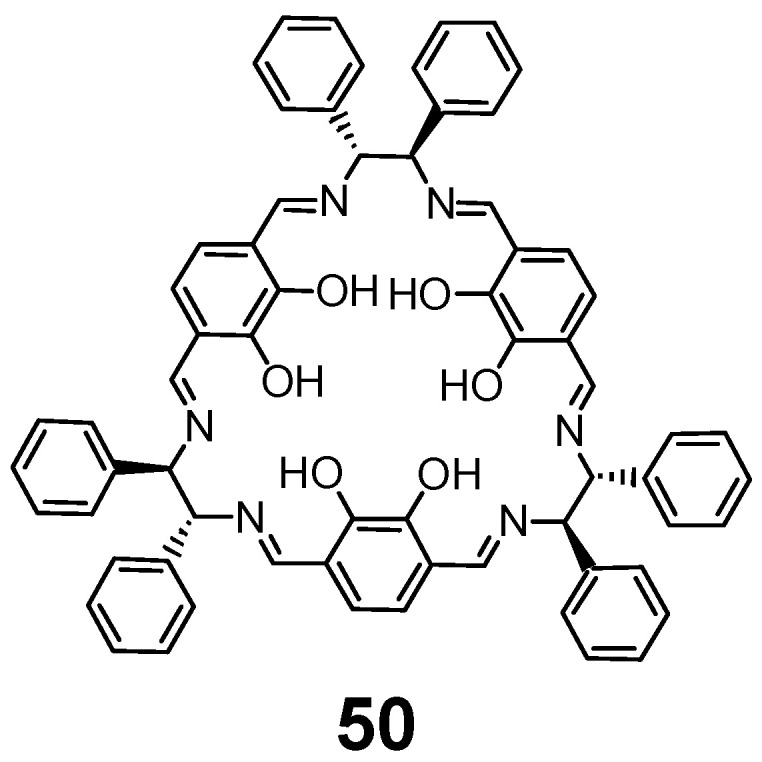
[3 + 3] macrocycle that is obtained in template condensation as a Zn_3_Er complex.

The chiral [3 + 3] imine **50** (Figure 21) is obtained in template condensation, where two types of metal ions are used simultaneously, thus three smaller transition metal ions, such as zinc(II) are bound in the three salen-type compartments, while the larger lanthanoid(III) ions occupy the central O_6_ cavity formed by deprotonated phenolic groups [121]. The Er(III) complex of this type exhibits remarkable single molecule magnet properties.

## 4. [4 + 4] Macrocycles

**Figure 22:**
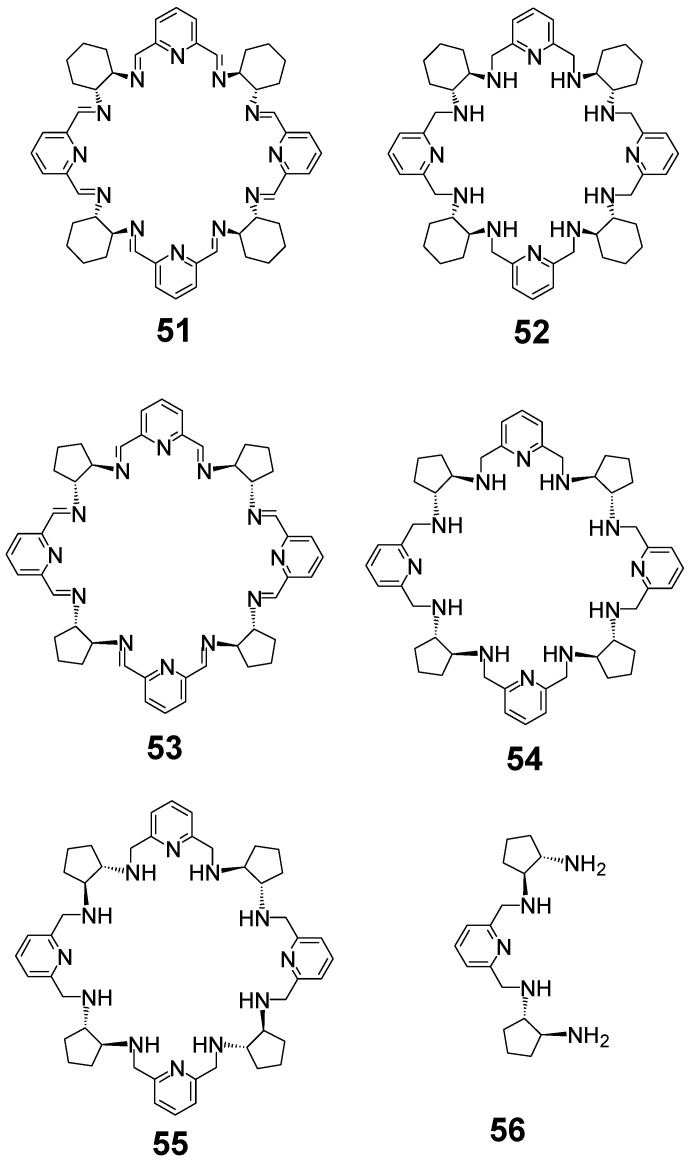
[4 + 4] macrocycles derived from DFP.

**Figure 23:**
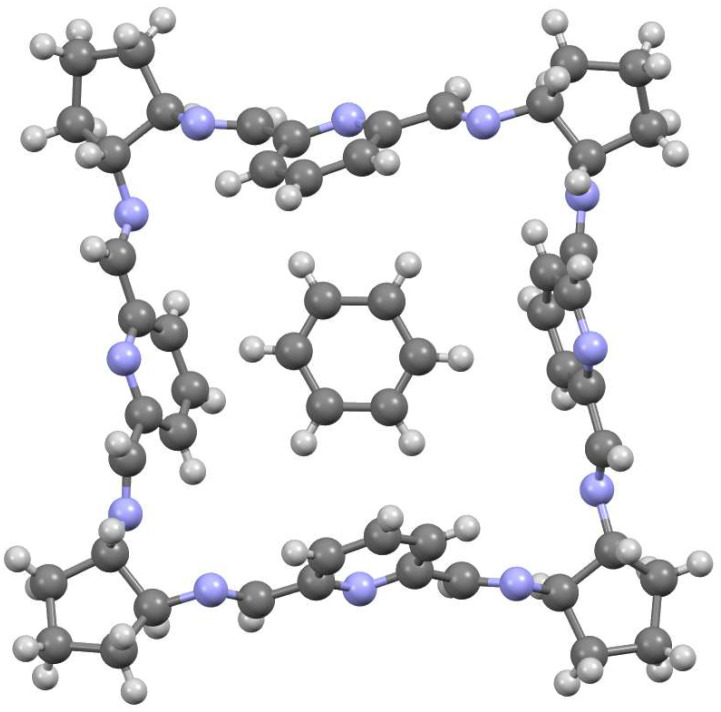
Molecular structure of **51** with benzene guest molecule.

The condensation of racemic DACH with DFP results in a mixture consisting mainly of *meso*-type achiral [2 + 2] macrocycle **10** and [4 + 4] macrocycle **51** of the alternating RRSSRRSS chirality of the cyclohexane fragments (Figure 22). The latter macrocycle can be separated and then reduced to the corresponding amine **52** [36]. In a similar reaction involving racemic DACP, only the [2 + 2] macrocycle is formed in methanol, but a mixture of macrocycles is formed in benzene, from which solvent *meso*-type achiral [4 + 4] imine **53** can be isolated and converted into a corresponding [4 + 4] amine **54** [122]. The crystal structure of **51** indicates a benzene guest molecule that is held in the center of the macrocycle via CH-π interactions (Figure 23). Amine **55** (all—(S) enantiomer), which is a homochiral isomer of amine **54**, may be isolated in small (5.6 %) yields by using gel permeation chromatography from the mixture of macrocyclic amines which are obtained by the reduction of the mixture of imines resulting from the condensation of enantiopure DACP and DFP (the main product being the [3 + 3] macrocycle). Alternatively, amine **55** may be obtained in high (52.2 %) yield in step-wise synthesis from the intermediate **56** and DFP [123]. Apart from *meso* amine **54** of RRSSRRSS chirality of diaminocyclohexane fragments and its homochiral isomer **55** of SSSSSSSS chirality, other isomers of RRRRSSSS (achiral) and RRRRRRSS (enantiopure) chirality may be obtained in step-wise synthesis via protection/deprotection strategy of linear intermediates [124].

**Figure 24:**
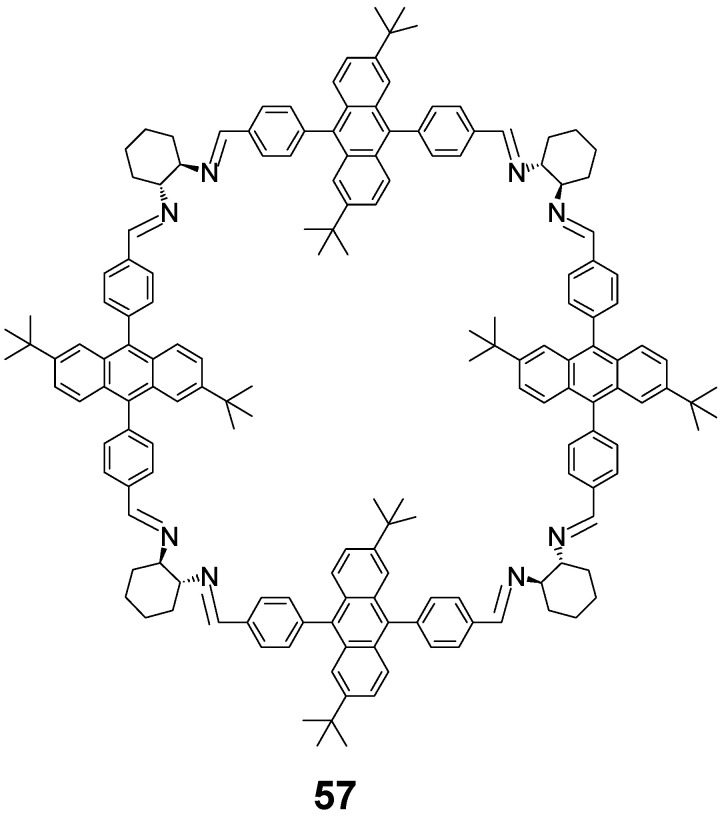
Giant [4 + 4] macrocycle 57 obtained in *p*-xylene-templated reaction.

The reaction of an extended linear dialdehyde with DACH in boiling toluene and in the presence of *p*-toluenesulfonic acid results in a mixture of [3 + 3] imine **44b** and [4 + 4] imine **57** (Figure 24). Heating the mixture of these macrocycles in *p*-xylene shifts the equilibrium completely towards the large macrocycle **57** [125]. The condensation of the substrates directly in *p*-xylene also led to **57** as the main product. These effects were not based on applying simply different reaction temperatures, which indicates a real template role of this solvent. The plausible template role of *p*-xylene, which is retained in the final product, may be based on the preorganization of substrates and/or stabilization of the [4 + 4] structure via CH-π and π-π interactions. The theoretical DFT structure of **57** suggests that the interior of this giant macrocycle is partly occupied by *tert*-butyl substituted 9,10-diphenylanthracene fragments. On the other hand, in the calculated structure of the amine counterpart of **57,** these fragments are perpendicular to the mean macrocycle plane. In this way, a nano-sized square box is formed with a distance between the anthracene units equal to 2.14 nm. Another interesting feature of **57** is the exceptionally strong amplitude of its electronic circular dichroism spectra.

**Figure 25:**
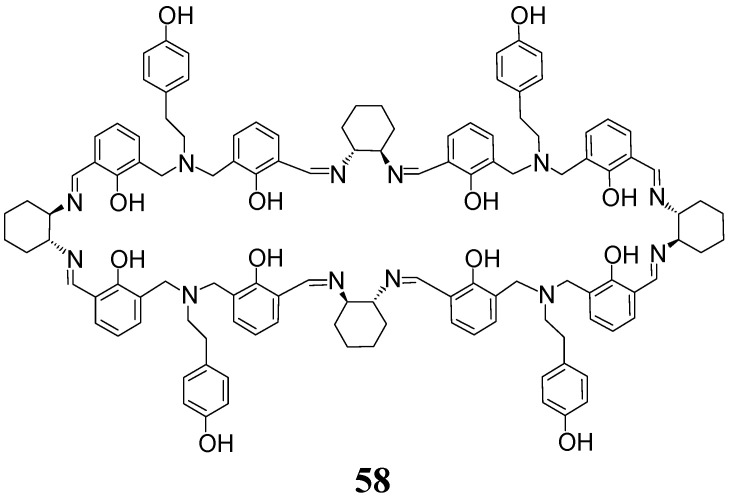
[4 + 4] imine obtained in anion-dependent template condensation with zinc(II).

A tetranuclear zinc(II) complex of [4 + 4] macrocycle **58** (Figure 25) can be obtained in a template reaction by using the same enantiopure DACH, the same aldehyde substrates and the same templating cation, which were used for the synthesis of dinuclear zinc(II) complex of the [2 + 2] macrocycle **27** [72]. In this system, an interesting secondary template effect of counterions is observed. Thus, the application of zinc(II) nitrate as a templating salt led to [2 + 2] imine, while the application of zinc(II) chloride as a templating salt led to [4 + 4] imine. This result indicates that not only the kind of the metal cation but also the kind of counter-anion may shift the equilibrium of imine condensation products towards a specific macrocycle.

## 5. [6 + 6] and [8 + 8] Macrocycles

**Figure 26:**
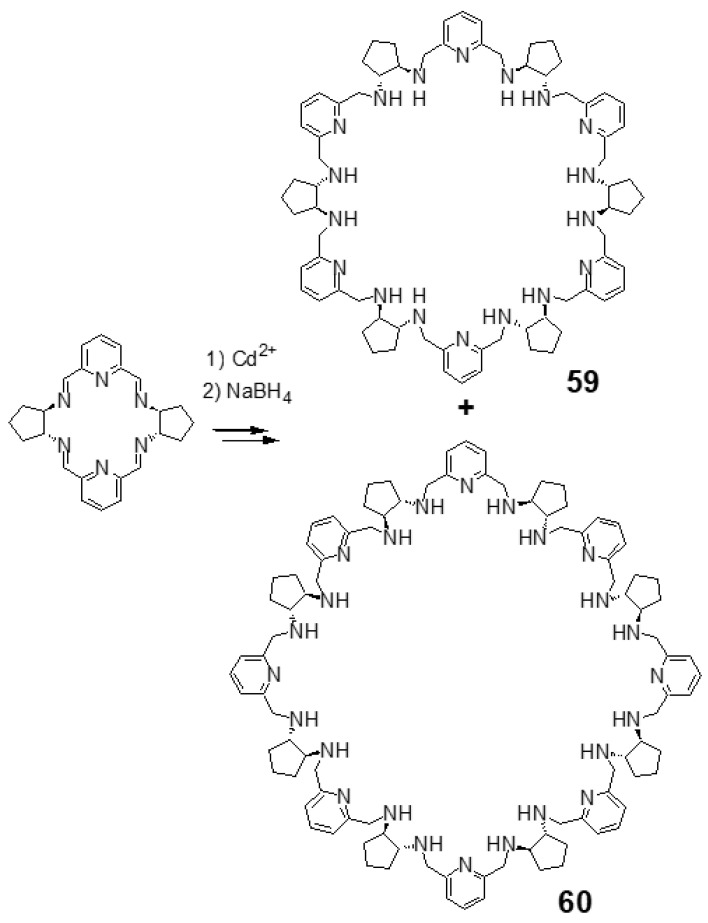
Expansion of the [2 + 2] macrocycle into giant *meso*-type macrocycles.

**Figure 27:**
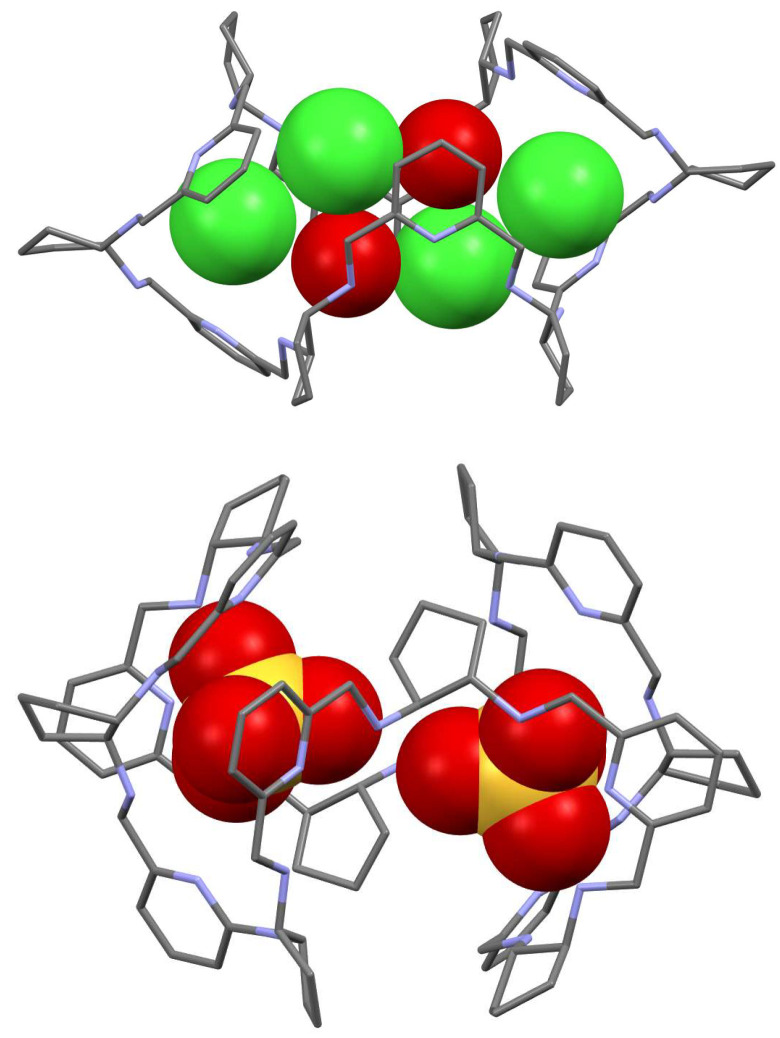
Top—structure of protonated [6 + 6] amine **59** embracing two chloride anions and two water molecules. Bottom—structure of protonated [8 + 8] amine **60** embracing two sulfate anions (chlorides, sulfates and water molecules are shown in spacefill representation, hydrogen atoms are omitted).

**Figure 28:**
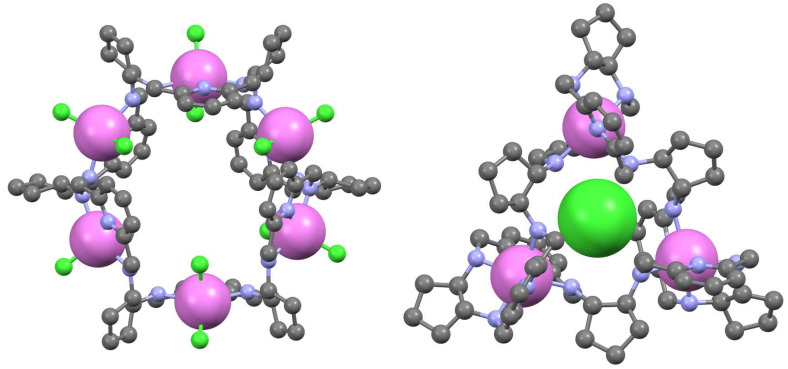
Molecular structures of the hexanuclear (**left**) and trinuclear (**right**) zinc(II) complexes of macrocycle **59**. Metal ions (violet) and the central chloride guest anion (green) are shown in spacefill representation, hydrogen atoms are omitted.

The reaction of meso-type [2 + 2] imine **10** with cadmium(II) chloride, followed by reduction with sodium borohydride leads to a giant [6 + 6] macrocyclic amine **59** as the main product and [8 + 8] amine **60** as the minor product (Figure 26) [122,126]. The X-ray crystal structure of the protonated **59** indicates a multiply folded macrocycle of a globular shape, which is a host molecule for four chloride anions and two solvent molecules (water or acetonitrile) embedded in the macrocycle (Figure 27). Similarly, the protonated form of the larger [8 + 8] macrocycle adopts globular multiply folded conformation and binds anion guest in the center (Figure 28). The neutral macrocycle **59** is predisposed for the binding of six transition metal cations, e.g., zinc(II) (Figure 28) and it adopts various conformations in these complexes [127]. It can also form a trinuclear zinc(II) complex, where the sections of the macrocycle wrap around the metal centers and the complex binds the chloride anion guest in the center (Figure 28).

**Figure 29:**
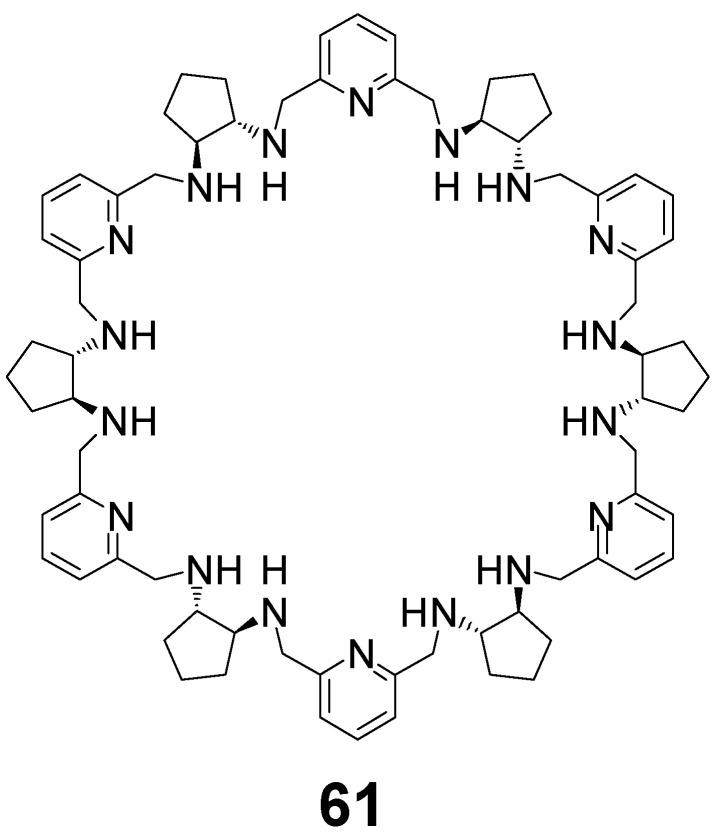
[6 + 6] homochiral macrocycle derived from DACP.

The direct condensation of enantiopure DACP with DFP followed by reduction with sodium borohydride leads to homochiral [6 + 6] amine **61** in trace amounts only (Figure 29) together with traces of [5 + 5] and [7 + 7] macrocycles. Macrocycle **61** can be isolated, however, in 10% yield by using gel permeation chromatography as a minor product accompanying the formation of homochiral [4 + 4] macrocycle **55** from the intermediate **56**.

**Figure 30:**
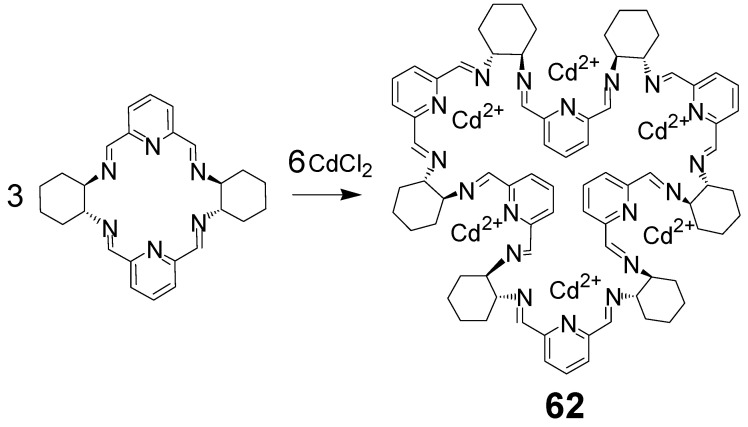
Macrocycle expansion reaction triggered by cadmium(II) template.

**Figure 31:**
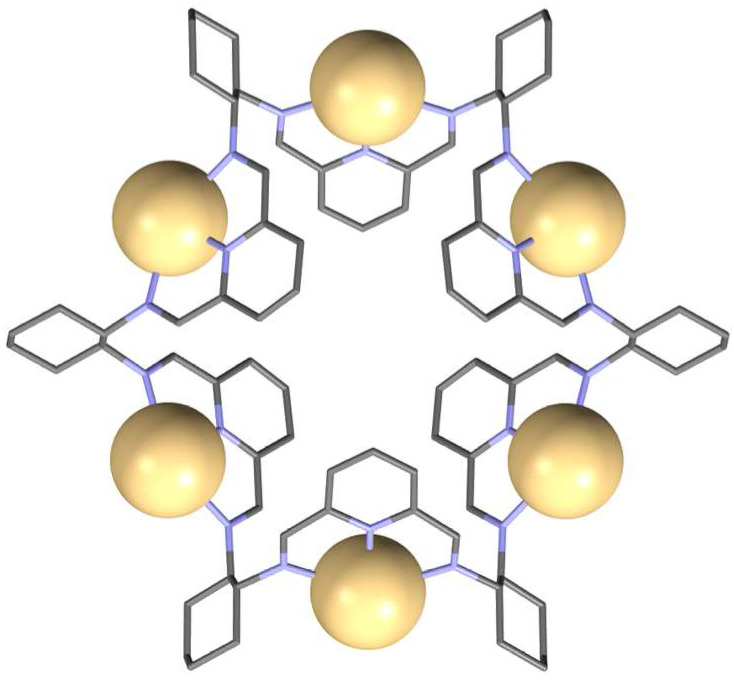
Molecular structure of hexanuclear Cd(II) complex of **62** (Cd atoms in spacefill representation, hydrogen atoms and chloride anions are omitted).

The X-ray crystal structures of the protonated **59** and its hexanuclear metal complexes show characteristic multiple folding of the macrocycle leading to the formation of six loops containing three nitrogen donor atoms each. This suggests that ring expansion of the [2 + 2] macrocycle under the influence of the templating Cd(II) ions is based on the reversible breaking of one of the imine bonds of the smaller macrocycle and reassembling of the linear fragments with the formation of six N3 sections for the coordination of six metal ions. Indeed, this hypothesis was confirmed in the case of an analogous reaction involving DACH instead of DACP derivatives [128]. In this case, the intermediate hexanuclear Cd(II) complex of the [6 + 6] imine macrocycle **62** was isolated and its X-ray crystal structure was determined (Figure 30 and Figure 31).

## 6. Macrocycles Containing Different Diamine Fragments

**Figure 32:**
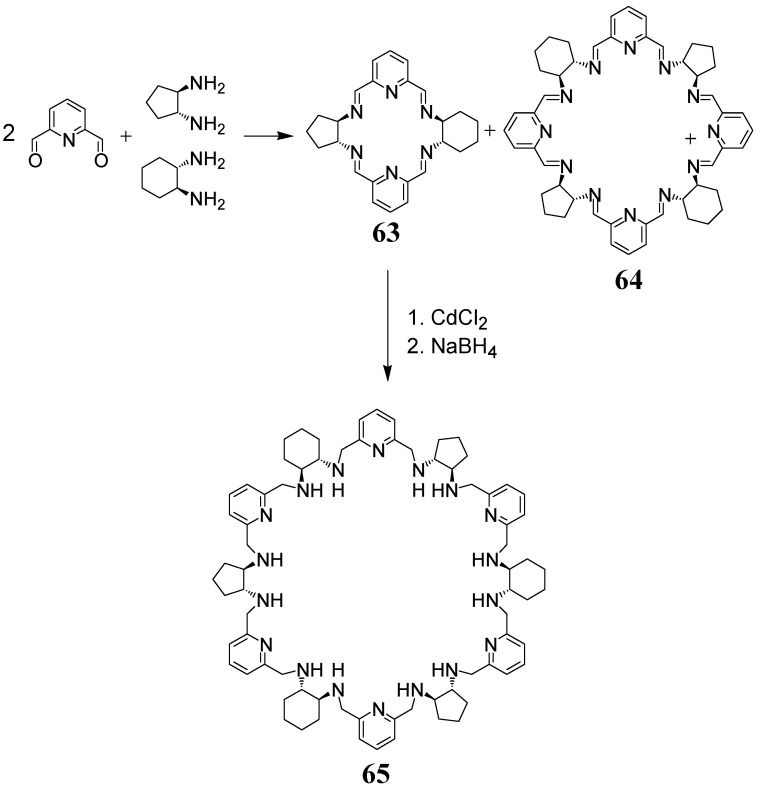
Macrocycles containing DACH and DACP rings.

The condensation of DFP with an equimolar mixture of two enantiopure diamines of opposite chirality, e.g., *trans*-(1S, 2S)- diaminocyclohexane and its cyclopentane analog *trans*-(1R, 2R)- diaminocyclopentane in methanol results in the isolation of [2 + 1 + 1] macrocycle **63** (Figure 32) [129]. In contrast, the same condensation in benzene allows for the isolation of a larger [4 + 2 + 2] macrocycle **64** (Figure 32). The above equimolar mixture of diamines may be regarded as a quasi-racemic analog of racemic DACH, while the macrocycles **63** and **64** may be regarded as analogs of the *meso*-type macrocycles **10** and **51,** respectively. It should be mentioned that the former two macrocycles are chiral and they can be obtained as pure enantiomers. This is because cyclohexane and cyclopentane rings are not equivalent, hence these macrocycles do not have the improper S_n_ symmetry axes present in the *meso*-type macrocycles. Similarly, a large [6 + 3 + 3] macrocycle **65** (Figure 32), which can be obtained in macrocycle expansion reaction of **63** under the template action of cadmium(II) ions followed by reduction to the amine form, is an enantiopure chiral analog of *meso*-type macrocycle **59** but has no S_6_ symmetry axis.

**Figure 33:**
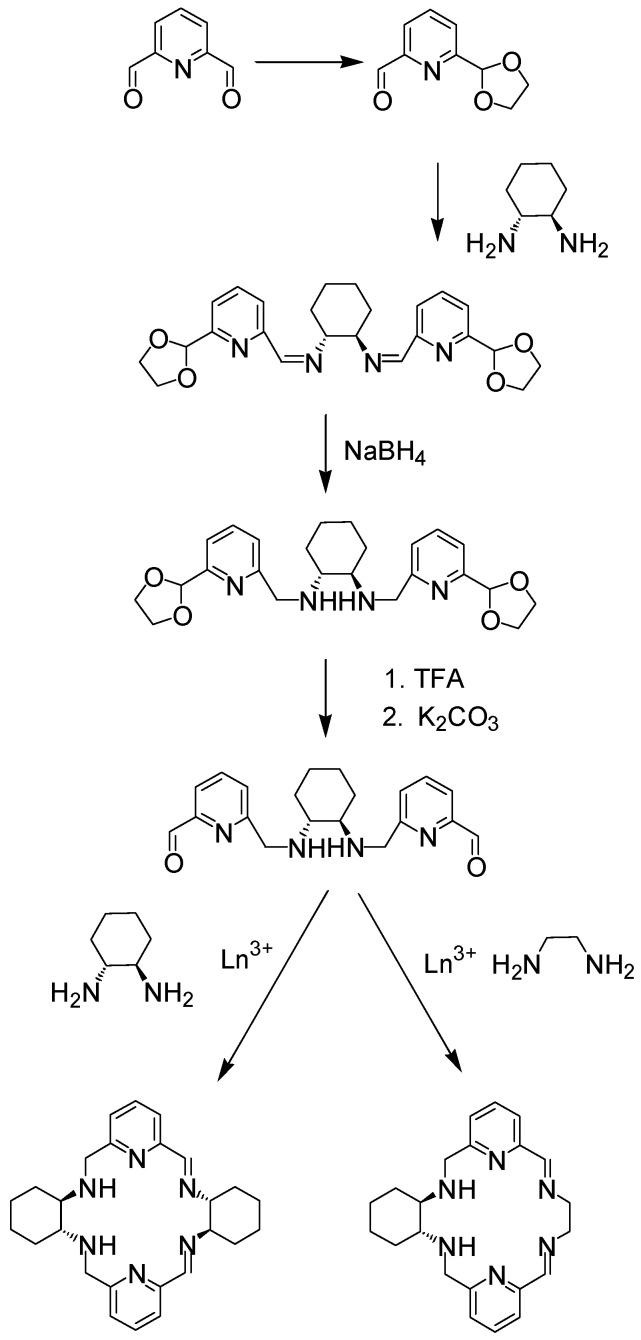
Step-wise synthesis of unsymmetrical imine/amine macrocycles.

Mixed imine/amine unsymmetrical [2 + 2] or [2 + 1 + 1] macrocycles can be obtained as lanthanoid(III) complexes in a multi-step synthesis involving protection/deprotection reactions (Figure 33) [130].

## 7. Conclusions

The [n + n] condensation of aromatic dialdehydes with chiral diamines is in many cases, a remarkably simple reaction that leads to defined imine macrocycles of various sizes and shapes. These expanded macrocycles, as well as their reduced amine counterparts, typically possess multiple donor atoms which predispose them to bind multiple metal ions. The cooperative catalytic activity of these ions combined with the enantiopure nature of the complexes of chiral macrocycles may lead to efficient enantioselective catalytic systems. The binding of multiple metal ions in close vicinity within the core of a large macrocycle may also result in magnetic interactions among these ions leading to new magnetic materials. The large macrocycles of this type often have the tendency to form supramolecular systems. In particular, they can function as hosts for the enantioselective binding of chiral organic guest molecules. This recognition process may find applications in the analysis and separation of enantiomers. The simplicity of the synthesis of the [n + n] imines and amines allows rather rational planning of new macrocycles of this type. Nevertheless, the final size of such condensation products depends not only on the kind of the used substrates, but sometimes it depends on the unexpected influence of the metal template, solvent, time and temperature of the condensation reaction, or chirality of the diamine. The effectiveness of this approach to the synthesis of macrocycles and the richness of the structures obtained so far undoubtedly indicates that in the future many new elaborate macrocyclic systems of this type will be obtained. In particular, further development of enantiopure multimetallic catalysts based on [n + n] macrocycles are desirable. Future progress in this field can be based on fine-tuning the chiral environment around the catalytic metal center, introducing additional steric hindrance and trying various metal centers. More advanced heterometallic macrocyclic complexes for enantioselective catalysis may be envisaged, in which the macrocycle will embrace different metal ions. The cooperative action of different metal centers with different chemical characters may potentially lead to new synergistic reactivity, different from that of the corresponding homometallic complexes. Yet another design that may lead to new catalytic effects may be based on mononuclear metal complexes of large [n + n] macrocycles. In such complexes, only part of the large macrocyclic cavity would be occupied by a catalytic metal ion, leaving the rest of the macrocyclic cavity for enantioselective binding of the guest substrate molecule. Moreover, in such a mononuclear complex of a large macrocyclic amine, there is an additional possibility for the cooperative action of Lewis acids and Brønsted base/acids in the catalytic cycle, similarly as has been observed in many metalloenzymes. Thus, the metal ion will act as a Lewis acid, while the part of the large macrocyclic amine that is not engaged in metal binding will act as a Brønsted base (or Brønsted acid if this part is partially protonated). Another prospective goal is to develop very large, shape-persistent [n + n] macrocycles of this kind for gas storage applications. For example, the introduction of additional basic substituents within the macrocycle may result in the preferential fixation of carbon dioxide.

## Data Availability

The data presented in this study are available on request from the corresponding author.

## Abbreviations

DACH	1,2-*trans*-diaminocyclohexane
DPEN	1,2-diphenylethylenediamine
DCAP	*trans*-1,2-diaminocyclopentane
DFP	2,6-diformylpyridine
SMM	Single Molecule Magnet
